# 

**Published:** 1857-04

**Authors:** 


*American Medical Association.*—The period for the annual convocation of this body, rapidly approaches. The session of this year will be held at Nashville, Tennessee, commencing Tuesday, May 7th. We bespeak for it a full attendance. For those who have the leisure, it will furnish a fine trip for recreation as well as for scientific improvement. The hospitality of our southern brethren is proverbial, and upon no occasion will it be more freely tendered than upon this gathering of the doctors.

At this meeting we hope the stereotyped course of electing the President of the Association from among the oldest practitioners of the place of meeting, because simply, he is the oldest and most prominent practitioner of the place, will be broken in upon. We long to see the gift of this office, considered the highest prize attainable by the profession of our country, and that the fact of its conference shall be evidence of the highest professional attainments and worth.

We hope the project of giving the association a permanent place of meeting, "a local habitation," will be defeated at this meeting. We say, let the association continue its old practice of migrating about the country. You will, by so doing, keep it out of the hands of cliques, and preserve it from old fogyism. Those who are desirous of seeing it collect a library might as well abandon the project at once. It can be better employed in the work of dispersion, than in that of collection. Let its funds be employed in the publication and dissemination of living knowledge, the productions of to-day, and not in the collection of dead and effete matters which would be accessible only to those living in the immediate vicinity of the library.

Dr. Robert C. Foster, Secretary of the Association, at Nashville, Tenn., desires the medical press throughout the Union, to copy the following resolutions at their earliest convenience; and we give them place in this connection. Societies must look to it, that they come with clean hands to the association, or they may receive uncivil treatment when they seek admittance at its deliberations:

Resolutions passed at the eighth meeting of the association, held at Philadelphia:

*Resolved*, That no State or local society shall hereafter be entitled to representation in this association, that has not adopted its code of ethics.

*Resolved*, That no State or local society that has intentionally violated or disregarded any article or clause in the code of ethics, shall any longer be entitled to representation in this body.

*Resolved*, That no organization or institution entitled to representation in this association, shall be considered in good standing, which has not adopted its code of ethics.

Resolution passed at the ninth meeting, held at Detroit:

*Resolved* That any new medical institution not heretofore represented in this body, be required to transmit to the secretary, with the credentials of its delegates, evidence of its existence, capacity and good standing."

---

As this is the season when many young men will make their exit from our schools of medicine and surgery to enter upon the duties of a professional life, wewould inform so many of them as may see and hear of this notice, that a fine opening may be had soon, by applying tn Dr. Nims, Homer, Calhoun County, Michigan. Dr. N. is desirous of finding a purchaser for his property, and will dispose of it on reasonable terms.

*Proposed Convention of Medical Editors.*—It is suggested that a convention of medical editors be held at the meeting of the American Medical Association, next month, at Nashville, "to deliberate upon all subjects pertaining to the support and progress of medicai periodlcal literature."

Evidently such a meeting, even were no definite measures adopted, would result in good. Whether, as has been proposed, it can be made to appear desirable for all the journals to adopt the cash in advance system, is very doubtful. Many of them are owned by secular publishers who prefer the credit system, which has certainly some advantages. Others are controlled by interests which require a large circulation, without much reference to pecuniary gain or loss. But at any rate something could be done toward an expression of opinion as to the proper course to be pursued, and many suggestions might be offered which would do away with some of the evils of journalism. The establishment of a black list strikes us not unfavorably. We could make a very handsome contribution to it on the start. Our journal is now one of the seniors. Without any change, other than one transfer of the editorship, it has been published from the same press for a period of twelve years. As a consequence we boast some subscribers who have taken it regularly all that time, *without paying a single dollar.*

We say this not reproachfully. Some of these men of long endurance are, as we hear, clever fellows, with short purses and shorter memories, and aside from them we have a noble list of prompt, paying subscribers. But it would be a good idea to punish these delinquent gentlemen by putting them on short rations of journalism, to say to them, as they forward their courteous notes enrolling their names as subscribers, without money, that they are unfortunately indebted to such a journal, and must pay up for that before getting another on credit.

The Boston Medical Journal suggests that something might be done, too, for those periodicals which are sustained not by original communications, which add nothing to the general wealth of the literature of medicine, but fill their pages with selections only. Whether anything could be done to put a stop to this piracy is a question, but we think there might. No journal should be established without some certainty of collaborators to maintain for it a fair character. Although, ordinarily, it is complimentary to be often quoted, yet we do know of some journals maintaining correspondents at such expense that we hardly think it fair to copy from that department of them, on the ground that they have a proprietary right in such expensive matter.

Let us see what can be done. It is hardly probable that we can make, as we would wish to do, the long journey to Nashville, but our southern brethren will be represented, and we of the north will give a fair hearing to their propositions.

